# Type B insulin resistance syndrome coexisting with aplastic anemia responsive to early immunosuppressive therapy

**DOI:** 10.1210/jcemcr/luag181

**Published:** 2026-07-13

**Authors:** Taro Fujisawa, Kazuhisa Takami, Mariko Horikawa, Masahiro Yokoyama, Tomoya Kawashima, Akiyoshi Takami

**Affiliations:** Department of Endocrinology and Metabolism/Diabetes Center, Central Japan International Medical Center, Minokamo 505-8510, Japan; Department of Diabetes, Endocrinology and Metabolism and Department of Rheumatology and Clinical Immunology, Gifu University Graduate School of Medicine, Gifu 501-1194, Japan; Department of Endocrinology and Metabolism/Diabetes Center, Central Japan International Medical Center, Minokamo 505-8510, Japan; Department of Endocrinology and Metabolism/Diabetes Center, Central Japan International Medical Center, Minokamo 505-8510, Japan; Department of Endocrinology and Metabolism/Diabetes Center, Central Japan International Medical Center, Minokamo 505-8510, Japan; Department of Endocrinology and Metabolism/Diabetes Center, Central Japan International Medical Center, Minokamo 505-8510, Japan; Department of Internal Medicine, Division of Hematology, Aichi Medical University School of Medicine, Nagakute, Aichi 480-1195, Japan

**Keywords:** type B insulin resistance syndrome, aplastic anemia, immunosuppressive therapy, autoimmune disease, insulin receptor autoantibody

## Abstract

Type B insulin resistance syndrome (IRS) is a rare autoimmune disorder characterized by severe insulin resistance caused by autoantibodies against the insulin receptor. Clinically, it presents with fluctuating hyperglycemia and hypoglycemia and is frequently associated with other autoimmune diseases. We report a case with the rarely reported combination of type B IRS and aplastic anemia. A 77-year-old woman with obesity presented with abrupt deterioration of glycemic control and bilateral lower extremity edema. Despite the administration of up to 300 units/day of insulin after hospitalization, hyperglycemia remained refractory. Markedly elevated insulin receptor autoantibody titers (85.9%) confirmed the diagnosis of type B IRS. Further evaluation for concomitant pancytopenia revealed aplastic anemia on bone marrow examination. Immunosuppressive therapy with cyclosporine and eltrombopag for aplastic anemia improved the hyperglycemia; however, early morning hypoglycemia, renal dysfunction associated with intravascular volume depletion, and persistent lower extremity edema remained unresolved. The addition of glucocorticoid therapy resulted in rapid clinical improvement and marked reduction in the insulin receptor autoantibody titers (8.6%). This case highlights the importance of early and appropriate immunomodulatory therapy to achieve remission in type B IRS, particularly in patients with multiple concomitant autoimmune diseases.

## Introduction

Insulin resistance syndrome (IRS) is a rare disorder that is characterized by severe insulin resistance due to impaired insulin receptor function or downstream signaling defects [1]. IRS is classified into type A, caused by genetic abnormalities, and type B, caused by autoantibodies against the insulin receptors [2, 3]. Although rare, the clinical characteristics of IRS have been increasingly elucidated. In Japan, a nationwide survey on IRS was published in 2020 [4], followed by updated classification and diagnostic criteria proposed by the Japan Diabetes Society in 2022 [5].

Type B IRS is characterized by profound insulin resistance mediated by circulating insulin receptor autoantibodies. Patients typically present with severe hyperglycemia requiring extremely high doses of insulin; paradoxical hypoglycemia may also occur, reflecting the fluctuating agonistic and antagonistic effects of these autoantibodies on the insulin receptor [6]. Type B IRS is frequently associated with autoimmune diseases, including systemic lupus erythematosus, autoimmune thyroid disease, Sjögren syndrome, and immune thrombocytopenia [4]. Importantly, remission of type B IRS has been reported following treatment of the concomitant autoimmune diseases [7, 8]. These findings highlight the importance of identifying the coexisting autoimmune conditions and initiating timely immunomodulatory therapy. Here, we report an unusual case of type B IRS complicated by aplastic anemia in which glucocorticoid therapy led to clinical remission.

## Case presentation

A 77-year-old woman with obesity was referred for rapidly worsening glycemic control over 3 months, with hemoglobin A1c increasing from 6.0% (IFCC: 41 mmol/mol) to 10.2% (IFCC: 88 mmol/mol) (reference range, 4.9-6.0% [IFCC: 30-41 mmol/mol]). Her body weight increased from 58 to 88 kg after retirement. Despite obesity, she had never been diagnosed with diabetes mellitus or received glucose-lowering therapy. Her family history was notable as both her mother and brother had type 2 diabetes mellitus. She reported no polyuria, polydipsia, nausea, or vomiting.

## Diagnostic assessment

At presentation, her height was 148.6 cm and body weight 79.2 kg (body mass index 35.9 kg/m^2^). Blood pressure was 121/60 mmHg, and pulse rate was 78 beats per minute. Physical examination revealed bilateral pitting edema, without acanthosis nigricans or features of Cushing syndrome, acromegaly, or lipodystrophy.

Laboratory tests showed marked hyperglycemia (random plasma glucose 166 mg/dL [SI: 9.2 mmol/L]; reference range, 70-110 mg/dL [SI: 3.9-6.1 mmol/L]) and hemoglobin A1c of 10.2% [IFCC: 88 mmol/mol]. Serum creatinine was 0.95 mg/dL [SI: 84.0 μmol/L] (reference range, 0.46-0.79 mg/dL [SI: 40.6-69.8 μmol/L]). Pancytopenia was present, with a white blood cell count of 2230/μL (reference range, 3300-8600/μL), hemoglobin of 10.5 g/dL (SI: 105 g/L) (reference range, 11.6-14.8 g/dL [SI: 116-148 g/L]), and platelet count of 71 000/μL (reference range, 158 000–348 000/μL). C-reactive protein was 0.33 mg/dL [SI: 3.3 mg/L] (reference range, 0.00-0.14 mg/dL [SI: 0.0-1.4 mg/L]), and there was no fever or clinical evidence of infection. Urinalysis revealed no proteinuria or hematuria (Table 1). Computed tomography of the chest and abdomen showed no evidence of malignancy or infection. The patient was admitted for further evaluation.

**Table 1:** Laboratory data at the initial visit

Biochemistry	Value	Reference range
TP	6.8 g/dL (68 g/L)	6.6-8.1 g/dL (66-81 g/L)
Alb	3.5 g/dL (35 g/L)	4.1-5.1 g/dL (41-51 g/L)
T-Bil	0.85 mg/dL (14.54 μmol/L)	0.4-1.5 mg/dL (3.42-20.52 μmol/L)
AST	18 IU/L	13-30 IU/L
ALT	13 IU/L	7-23 IU/L
γ-GTP	26 IU/L	9-32 IU/L
LDH	160 IU/L	123-222 IU/L
Cre	0.95 mg/dL (84.0 μmol/L)	0.46-0.79 mg/dL (40.6-69.8 μmol/L)
BUN	13.8 mg/dL (4.93 mmol/L)	8.0-20.0 mg/dL (2.86-7.14 mmol/L)
eGFR	43.6 mL/min/1.73 m^2^	>60 mL/min/1.73 m^2^
UA	4.3 mg/dL (255.8 μmol/L)	2.6-5.5 mg/dL (220.1-463.9 μmol/L)
Sodium	137 mEq/L (137 mmol/L)	138-145 mEq/L (138-145 mmol/L)
Potassium	4.6 mEq/L (4.6 mmol/L)	3.6-4.8 mEq/L (3.6-4.8 mmol/L)
chloride	104 mEq/L (104 mmol/L)	101-108 mEq/L (101-108 mmol/L)
Calcium	9.5 mg/dL (2.37 mmol/L)	8.8-10.1 mg/dL (2.20-2.52 mmol/L)
CRP	0.33 mg/dL (3.3 mg/L)	0.00-0.14 mg/dL (0.0-1.4 mg/L)
Plasma glucose	166 mg/dL (9.2 mmol/L)	70-110 mg/dL (3.9-6.1 mmol/L)
HbA1c	10.2% (88 mmol/mol)	4.9-6.0% (30-41 mmol/mol)
C-peptide	8.1 ng/mL (2.7 nmol/L)	0.6-1.8 ng/mL (0.2-0.6 nmol/L)
**Blood count**		
WBC	2230/μL	3300-8600/μL
NEUT	52.0%	41.0-59.0%
LYMPH	36.0%	26.0-46.6%
MONO	11.0%	2.3-7.7%
EOSINO	0.0%	0.0-5.0%
BASO	1.0%	0.0-1.0%
RBC	320 × 10^4^/μL	386-492 × 10^4^/μL
Hb	10.5 g/dL (105 g/L)	11.6-14.8 g/dL (116-148 g/L)
Hct	31.6%	35.1-44.4%
Plt	7.1 × 10^4^/μL	15.8-34.8 × 10^4^/μL
**Urinalysis**		
Protein	Negative	Negative
Glucose	Negative	Negative
Blood	Negative	Negative

Values in parentheses are International System of Units.

Abbreviations: Alb, albumin; ALT, alanine aminotransferase; AST, aspartate aminotransferase; BUN, blood urea nitrogen; Cre, creatinine; CRP, C-reactive protein; eGFR, estimated glomerular filtration rate; Hb, hemoglobin; Hct, hematocrit; LDH, lactate dehydrogenase; Plt, platelets; RBC, red blood cell count; T-Bil, total bilirubin; TP, total protein; UA, uric acid; WBC, white blood cell count; γ-GTP, γ-glutamyl transpeptidase.

**Figure 1:**
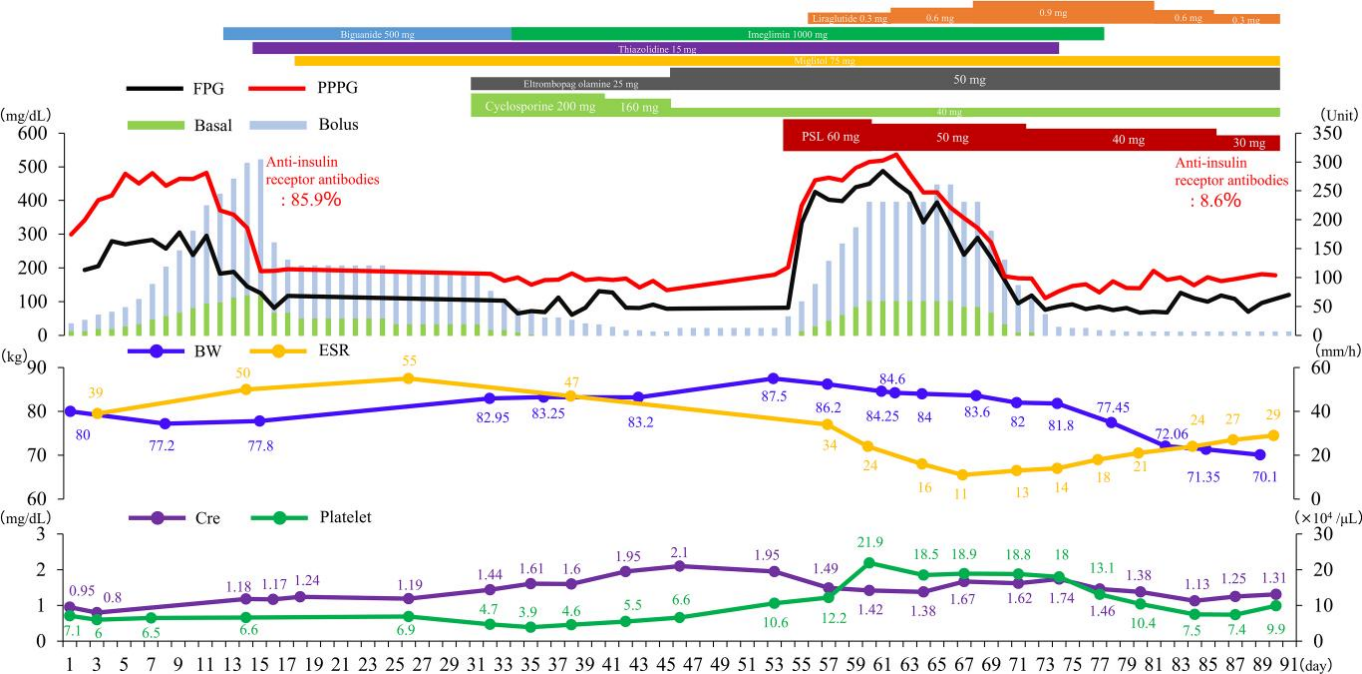
Clinical course and treatment timeline. Severe hyperglycemia persisted despite high-dose insulin therapy. The addition of metformin, thiazolidinedione, and miglitol reduced insulin requirements. Treatment with cyclosporine and eltrombopag for aplastic anemia improved hyperglycemia. However, early morning hypoglycemia, renal dysfunction, which was considered multifactorial, including inflammatory intravascular volume depletion and cyclosporine-associated nephrotoxicity, and weight gain due to lower extremity edema persisted. Prednisolone was initiated at 60 mg/day; marked steroid-induced hyperglycemia was observed initially, and liraglutide was introduced and titrated to 0.9 mg/day. Approximately 2 weeks after glucocorticoid initiation, glycemic control improved, accompanied by the resolution of edema-related weight gain and improvement in renal function. By the time prednisolone was tapered to 30 mg/day, insulin receptor autoantibody titers had normalized (8.6%). BW, body weight; Cre, creatinine; ESR, erythrocyte sedimentation rate; FPG, fasting plasma glucose; PPPG, postprandial plasma glucose; PSL, prednisolone.

Despite initiation of insulin therapy (insulin lispro 6 units before each meal and insulin glargine 8 units at bedtime; total 20 units/day, approximately 0.3 units/kg/day), a calorie-restricted diet (1440 kcal/day), and progressive escalation of insulin doses to 300 units/day, severe hyperglycemia persisted, with fasting plasma glucose levels of 200-300 mg/dL (SI: 11.1-16.7 mmol/L) and postprandial levels of 400-500 mg/dL (SI: 22.3-27.9 mmol/L) (Fig. 1). Serum C-peptide was elevated at 8.1 ng/mL (SI: 2.7 nmol/L) (reference range, 0.6-1.8 ng/mL [SI: 0.2-0.6 nmol/L]), indicating severe insulin resistance. Serum insulin-like growth factor-1 (IGF-1) was decreased at 14 ng/mL (SI: 1.8 nmol/L) (reference range: 49-158 ng/mL [SI: 6.4-20.7 nmol/L]). Insulin receptor autoantibodies, measured using a radioreceptor assay at SRL Inc. (Tokyo, Japan), showed a positive inhibition rate of 85.9% (reference range: not applicable), confirming type B IRS (Table 2).

**Table 2:** Additional laboratory data after admission (under fasting)

Immunological evaluation	Value	Reference range
ESR	33 mm/h	3-15 mm/h
ANA	1:320	<1:40
Anti-dsDNA Ab	4.2 IU/mL	0.0-12.0 IU/mL
Anti-SS-A Ab	≥1200 U/mL	<10.0 U/mL
Anti-SS-B Ab	3.7 U/mL	<10.0 U/mL
TPOAb	6.11 IU/mL	<5.61 IU/mL
TgAb	393 IU/mL	<4.11 IU/mL
TRAb	<0.8 IU/L	<2.0 IU/L
*H. Pylori* Ab	3 U/mL	<10 U/mL
GAD Ab	<5 U/mL	<5 U/mL
IA-2 Ab	<0.6 U/mL	<0.6 U/mL
Insulin receptor autoantibody	Positive (inhibition rate 85.9%)	Not applicable
Thrombopoietin	3.73 fmol/mL (3.73 pmol/L)	≤0.68 fmol/mL (≤0.68 pmol/L)
sIL-2R	793 U/mL	121-613 U/mL
**Endocrinological evaluation**		
TSH	5.05 μIU/mL (5.05 mIU/L)	0.50-5.00 μIU/mL (0.50-5.00 mIU/L)
Free T3	1.99 pg/mL (4.62 pmol/L)	2.30-4.00 pg/mL (3.53-6.14 pmol/L)
Free T4	1.44 ng/dL (18.5 pmol/L)	0.90-1.70 ng/dL (11.6-21.9 pmol/L)
ACTH	17.7 pg/mL (3.90 pmol/L)	7.2-63.3 pg/mL (1.6-14.0 pmol/L)
Cortisol	9.4 μg/dL (259 nmol/L)	3.7-19.4 μg/dL (102-535 nmol/L)
FSH	44.77 mIU/mL (44.77 IU/L)	≤157.79 mIU/mL (≤157.79 IU/L)
LH	16.08 mIU/mL (16.08 IU/L)	5.72-64.31 mIU/mL (5.72-64.31 IU/L)
Testosterone	0.57 ng/mL (2.0 nmol/L)	0.12-0.31 ng/mL (0.42-1.07 nmol/L)
Progesterone	<1.4 ng/mL (4.45 nmol/L)	<0.2 ng/mL (0.64 nmol/L)
Estradiol	23 pg/mL (84 pmol/L)	≤39 pg/mL (≤143 pmol/L)
GH	0.49 ng/mL (0.49 μg/L)	0.13-9.88 ng/mL (0.13-9.88 μg/L)
IGF-1	14 ng/mL (1.8 nmol/L)	49-158 ng/mL (6.4-20.7 nmol/L) (age-adjusted reference range)

Values in parentheses are International System of Units. All hormonal measurements were obtained from early morning fasting blood samples at rest.

Abbreviations: ACTH, adrenocorticotropic hormone; ANA, anti-nuclear antibody; Anti-dsDNA Ab, anti-double stranded DNA antibody; Anti-SS-A Ab, anti-SS-A antibody; Anti-SS-B Ab, anti-SS-B antibody; ESR, erythrocyte sedimentation rate; FSH, follicle-stimulating hormone; GAD Ab, glutamic acid decarboxylase antibody; GH, growth hormone; *H. pylori* Ab, *Helicobacter pylori* antibody; IA-2 Ab, insulinoma-associated protein 2 antibody; IGF-1, insulin-like growth factor-1; LH, luteinizing hormone; sIL-2R, soluble IL-2 receptor; TgAb, thyroglobulin antibody; TPOAb, thyroid peroxidase antibody; TSH, thyroid-stimulating hormone.

Bone marrow examination demonstrated hypocellularity with fatty replacement, consistent with aplastic anemia. Immunological evaluation revealed anti-SS-A antibody positivity, and ophthalmological findings supported the diagnosis of Sjögren syndrome, although the patient had no clinical signs of the disease. Anti-thyroglobulin and anti–thyroid peroxidase antibodies were positive. Thyroid function showed mildly elevated thyroid-stimulating hormone at 5.05 μIU/mL [SI: 5.05 mIU/L] (reference range, 0.50-5.00 μIU/mL [SI: 0.50-5.00 mIU/L]) with normal free thyroxine, consistent with subclinical hypothyroidism not requiring treatment. Upper endoscopy was unremarkable, and testing for *Helicobacter pylori* was negative.

## Treatment

Metformin (500 mg/day), pioglitazone (15 mg/day), and miglitol (75 mg/day) were added, resulting in partial improvement in hyperglycemia and reduction in insulin requirements. Cyclosporine (200 mg/day) and eltrombopag (25 mg/day) were initiated for aplastic anemia; however, early morning hypoglycemia, renal dysfunction, and edema persisted. Therefore, prednisolone was started at 60 mg/day (0.8 mg/kg/day).

Glucocorticoid therapy initially exacerbated the hyperglycemia, requiring intensification of insulin therapy. Liraglutide was introduced and titrated to 0.9 mg/day. Sjögren syndrome remained asymptomatic. The patient remained euthyroid and did not require treatment for autoimmune thyroiditis. Intermittently scanned continuous glucose monitoring (isCGM) was commenced after initiating treatment for aplastic anemia.

## Outcome and follow-up

**Figure 2:**
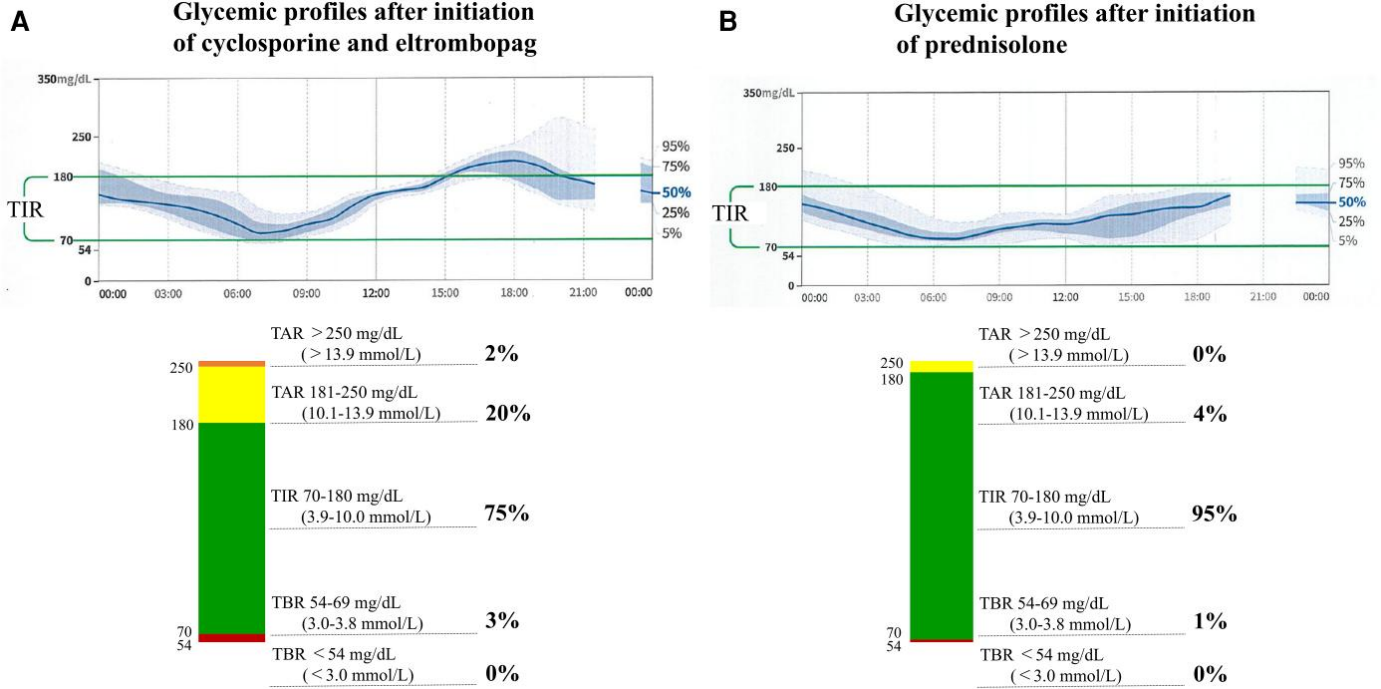
Glycemic profiles assessed by intermittently scanned continuous glucose monitoring. (A) Fourteen-day glycemic profile starting on day 8 after the initiation of cyclosporine and eltrombopag, demonstrating recurrent early morning hypoglycemia. (B) Fourteen-day glycemic profile starting on day 14 after the initiation of prednisolone, showing marked reduction in early morning hypoglycemia and an increase in TIR. TAR, time above range; TBR, time below range; TIR, time in range.

Oral glucose-lowering agents reduced insulin requirements and improved hyperglycemia, and this trend was further enhanced after the initiation of cyclosporine and eltrombopag. However, isCGM revealed early morning hypoglycemia, with nadir glucose levels of 60 mg/dL (SI: 3.3 mmol/L), accompanied by hunger (Fig. 2). Hypoglycemia was not present during insulin-only therapy, but only emerged after starting combination therapy. Renal dysfunction developed during treatment, with serum creatinine increasing to 2.10 mg/dL (SI: 185.6 μmol/L), accompanied by edema and weight gain up to 87.5 kg. This deterioration was likely multifactorial, including cyclosporine-associated nephrotoxicity and inflammatory intravascular volume depletion.

Following the initiation of prednisolone, blood glucose levels initially increased; however, within 2 weeks after initiation, hypoglycemia resolved, edema and renal dysfunction improved, and glycemic variability stabilized, enabling the tapering of insulin and liraglutide. Although insulin requirements had declined prior to glucocorticoid therapy, prednisolone was associated with the resolution of residual clinical manifestations.

These improvements were sustained during tapering of prednisolone. At discharge, body weight had decreased to 70.1 kg, and serum creatinine had improved to 1.31 mg/dL (SI: 115.8 μmol/L) (Figs. 1 and 2).

Immediately before discharge, when the prednisolone dose was reduced to 30 mg/day, the insulin receptor autoantibody inhibition rate decreased to 8.6%, which was within the normal range. Prednisolone is currently being tapered at 2.5-5.0 mg per month, with the goal of maintaining the lowest effective dose to sustain clinical remission.

## Discussion

In the present case, a 77-year-old woman with severe obesity experienced persistent marked hyperglycemia despite receiving up to 300 units/day of insulin, indicating profound insulin resistance. The diagnosis of type B IRS was confirmed by elevated levels of insulin receptor autoantibodies, while aplastic anemia was identified on bone marrow examination.

Type B IRS is characterized by severe insulin resistance mediated by insulin receptor autoantibodies [1]. A hallmark clinical feature of type B IRS is the coexistence of hyperglycemia and hypoglycemia [5]. Hyperglycemia results from impaired insulin signaling due to receptor dysfunction [1], whereas the pathogenesis of hypoglycemia remains incompletely understood; both inhibitory and stimulatory insulin receptor autoantibodies have been implicated [9]. Hypoglycemia is clinically significant, and fatal cases due to hypoglycemia in patients with type B IRS have been reported [10], underscoring the importance of preventive strategies. In this case, hypoglycemia emerged after starting combination therapy with oral glucose-lowering agents and reduced insulin requirements. Although this suggests the contribution of therapy, the mechanism is likely multifactorial, and involvement of insulin receptor autoantibodies cannot be excluded. Improvement after glucocorticoid therapy, with a marked reduction in autoantibody titers, supports the role of antibody-mediated mechanisms in glycemic instability. The patient also exhibited markedly reduced IGF-1 levels, consistent with prior reports suggesting dysregulation of the insulin–growth hormone–IGF-1 axis in severe insulin resistance [11].

Another important feature of type B IRS is its frequent association with autoimmune diseases [5]. A nationwide Japanese survey reported that 57% of patients with type B IRS had at least one concomitant autoimmune disorder [4]. In the present case, aplastic anemia, Sjögren syndrome, and autoimmune thyroid disease were identified. Cases of type B IRS complicated by aplastic anemia have rarely been described in the literature, suggesting an expansion of the clinical spectrum of autoimmune diseases associated with insulin receptor autoimmunity. Acquired aplastic anemia is widely recognized as an immune-mediated bone marrow failure syndrome. Accumulating evidence indicates that autoreactive cytotoxic T lymphocytes suppress hematopoiesis through cytokine-mediated mechanisms involving interferon-γ and tumor necrosis factor-α, leading to apoptosis and functional exhaustion of hematopoietic stem and progenitor cells [12, 13]. Moreover, the clinical efficacy of immunosuppressive therapy supports the autoimmune pathogenesis of acquired aplastic anemia [12]. Taken together, the coexistence of type B IRS and aplastic anemia in this patient was unlikely to be coincidental and may instead reflect shared underlying systemic immune dysregulation.

Management of type B IRS includes both metabolic control and immunosuppression. Glycemic management is often challenging because of profound insulin resistance [14]. Although insulin-sensitizing agents such as metformin and glucagon-like peptide-1 receptor agonists may provide partial benefit [15, 16], conventional glucose-lowering therapy alone is frequently insufficient. Recombinant human IGF-1 has also been reported as a therapeutic option in selected cases [17]. Hypoglycemia may also be difficult to manage and may require prolonged intravenous glucose infusion [18].

In contrast, immunosuppressive therapy is critical, especially in severe or refractory cases. In the Japanese survey, immunosuppressive therapy was administered to 47% of patients [4]. Although no standardized treatment strategy has been established and therapeutic responses vary, disease activity often parallels that of concomitant autoimmune disorders. Spontaneous remission has been reported in approximately one-third of patients within 11-48 months [2]. Nevertheless, when disease activity of concomitant autoimmune conditions is high or when patients present with severe and difficult-to-control hyperglycemia or hypoglycemia, early initiation of immunosuppressive therapy is critical. In the present case, cyclosporine and eltrombopag alone were insufficient to control the hypoglycemia, renal dysfunction, and edema. Early addition of glucocorticoid therapy resulted in marked clinical and immunological improvement. The optimal duration of glucocorticoid therapy remains uncertain, and although antibody titers were not measured before glucocorticoid initiation, an earlier decline cannot be excluded. Serial measurement may be useful for monitoring disease activity; however, its routine use is limited by restricted availability and cost considerations. The temporal association between the decline in the autoantibody inhibition rate and improvement in glycemic instability further supports the pathogenic role of insulin receptor autoantibodies in this case.

In conclusion, this report describes a case with rare coexistence of type B IRS and aplastic anemia. Although treatment directed solely at aplastic anemia was insufficient to achieve clinical remission, early initiation of glucocorticoid-based immunosuppressive therapy resulted in the resolution of clinical manifestations and immunological remission. This case highlights the importance of early immunomodulatory intervention in patients with refractory clinical features associated with concomitant autoimmune diseases.

## Learning points

- Type B IRS should be suspected in patients with severe insulin resistance accompanied by recurrent hypoglycemia. Definitive diagnosis requires measurement of insulin receptor autoantibodies.
- Because type B IRS is frequently associated with autoimmune diseases, management of concomitant autoimmune conditions—including aplastic anemia—may contribute to remission.
- Although spontaneous remission has been reported, early immunomodulatory therapy should be considered in patients with severe or refractory disease.

## Data Availability

Original data generated and analyzed during this study are included in this published article.

## Abbreviations

IGF-1: insulin-like growth factor-1
IRS: insulin resistance syndrome
isCGM: intermittently scanned continuous glucose monitoring
